# Nation-Wide Dissemination of a Digital Checklist to Improve Work Environment in the Eldercare Sector in Denmark

**DOI:** 10.3389/fpubh.2020.502106

**Published:** 2020-12-03

**Authors:** Pernille Kold Munch, Marie Birk Jørgensen, Helene Højberg, Charlotte Diana Nørregaard Rasmussen

**Affiliations:** ^1^National Research Centre for the Working Environment, Copenhagen, Denmark; ^2^Health and Safety, Municipality of Copenhagen, Copenhagen, Denmark

**Keywords:** re-aim, campaign, workplace, reach, adoption, maintenance, implementation, evaluation

## Abstract

In this study, we evaluated the dissemination of a digital checklist for improving implementation of work environment initiatives in the Danish eldercare sector. We evaluated the impact of the checklist using the RE-AIM framework. Initiated in 2016, researchers and relevant stakeholders were responsible for disseminating the checklist to all workplaces in the eldercare sector in Denmark through a national campaign. The checklist guided the user to define an action plan to implement, and the checklist covered 11 implementation concept points that should be addressed to reach full implementation of the action in focus. One year after the launch of the campaign almost all municipalities in Denmark had visited the website hosting the checklist (96%), 17% of individual workers within the eldercare responding to a union survey was reached, 4% (*n* = 199) of all eligible eldercare workplaces in Denmark and 8% of all nursing homes had adopted the checklist. Of the workplaces that used the checklist, 46% typed an action in the checklist. There were 13% of the first time users that used the checklist twice and 29% of the actions were revised (maintenance) after working with the implementation. Finally, the workplaces that had used the checklist showed a higher prioritization of work environment compared to workplaces not using the checklist both at baseline and at follow up. In conclusion, this study employing various strategies, including a 1-year national campaign to disseminate a checklist shows potential to impact implementation of work environment initiatives in the Danish eldercare sector. While dissemination is satisfactory and likely to increase further with time, more efforts is needed to ensure maintenance.

## Introduction

Currently many countries are facing shortage of healthcare workers, and the trends are forecasted to continue (1). In addition, the demographic changes in the Western World mean that there will be more elderly people with need for care. Thus, there is a great need for healthcare workers being healthy and fit to care for the elderly. Both the physical and psychosocial work environment is important factors for maintaining a healthy and fit workforce (2). Thus, several initiatives have been introduced in Denmark to improve the work environment among eldercare workers (3–6). The effect of work environment initiatives has often been reported in the scientific literature (7) and effective evidence-based work environment intervention studies among eldercare workers are available (3, 4, 6). A major limitation is the emphasis on efficacy and effectiveness of the initiatives, with little attention paid to the overall public health impact, which takes into account the dissemination potential of the initiative—the extent to which the initiative can be delivered to a large number of people and sustained over time.

There is a huge challenge in translation of policies and research knowledge into practice (8). Many factors can influence whether the translation of research knowledge into practice is successful and whether policies or evidence based practices are accepted and used by the target users (9). Dissemination of research findings is an important step to bridge the gap between research and practice. Effective dissemination strategies include formative research to customize dissemination strategies to fit audience needs and preferences (10). Distribution strategies should focus on ensuring that messages and materials from research reach intended audiences by use of multicomponent dissemination strategies, e.g., mailings, websites, publications, webinar or in-person presentations, interpersonal connections, and mass media among others (10, 11). To be most effective, distribution should engage the channels that intended audiences already trust and access for information (10). Thus, in a recent initiative in Denmark, a checklist was developed in collaboration with key stakeholders, to guide the implementation of work environment initiatives in eldercare sector workplaces. Given that the checklist is sector-specific for work environment initiatives, and developed through systematic collaboration between research and practice, it is likely to have high utility and impact. However, to evaluate the impact it is important to examine when, why, and how the checklist is spread to the Danish eldercare sector, in particular nursing homes and homecare.

A commonly used framework in the evaluation of public health impact of health promotion interventions is the RE-AIM framework (12–15). The RE-AIM model offers a useful framework for assessing the overall public health impact (12). The model focuses on five evaluation dimensions: reach (i.e., proportion of the target population that participated), efficacy (i.e., success rate at changing desired outcomes), Adoption (i.e., proportion of target settings involved), implementation (i.e., extent to which the program was delivered as intended), and maintenance (i.e., extent to which the program becomes a part of the routine) (12). The RE-AIM framework has been used in various fields including the evaluation of clinical guidelines implementation (12, 16, 17). To expand knowledge in the area on implementation and dissemination of work environment initiatives the five dimensions in the RE-AIM framework will be investigated.

To ensure effective interventions and to improve the work environment in the future, knowledge of the dissemination strategies, the workplace adoption, the reach of employees, the implementation and maintenance of these initiatives are important. The aim of this study is therefore to evaluate the dissemination and reach, adoption, implementation, maintenance, and effectiveness of the checklist to improve implementation of work environment initiatives among eldercare workers in Denmark.

## Materials and Methods

### Study Setting and Population

The study setting is the eldercare sector in Denmark, and more specifically nursing homes and homecare settings. In Denmark, there are ~5,000 workplaces within the eldercare sector that employ about 100,000 eldercare workers in total.

### Dissemination

#### Dissemination Object—A Digital Checklist

We developed a digital checklist in collaboration with key stakeholders, which was connected to a specific developed website (can be accessed on *www.MEDvirknu.dk*). The users (primarily the occupational health and safety (OHS) groups) can use it in their work environment practice when implementing new routines, projects, or initiatives (termed an “action” in the checklist) to improve the work environment. The development and content of the checklist has earlier been described in details (18, 19). In brief, the checklist is an interactive digital platform and has 11 implementation concept points (implementation concept points related to implementation, e.g., involvement of relevant employees, supervisor support, allocation of resources, etc.). First, the user has to log in with their affiliation, and then choose the action they want to implement. The next step is to go through the checklist, check the implementation concept points they have covered already and pick the implementation concept point in the checklist which they want to focus on to fulfill implementation. After having gone through all the points of the checklist, it is possible to print a diploma, tips and a letter. The diploma works as a process document and includes the work environment action, the implementation concept points already covered, and the implementation concept point to focus further on to make sure the implementation of the work environment action is fulfilled. A supplement to the diploma is tips covering how to begin and continue the work with the chosen implementation concept point and the letter describes in detail the checklist, the work environment action and the implementation concept points and is used for circulation in the management or among other local stakeholder groups to inform them of the action and the implementation progress.

#### Dissemination Strategy

To promote the checklist, we planned a national campaign, focusing at the eldercare sector and specifically the nursing homes. The campaign consisted of digital elements (videos, newsletters, social media, etc.), oral presentations (workshops, train-the-trainer, training, and conferences) and paper elements (printed checklist as postcards, magnets, letters, and magazines). The researchers and stakeholders primarily drove the campaign. The campaign was running for 1 year, starting the 4th of September 2017 ending 3rd of September 2018. The website remains open, regardless of the ended campaign.

### Design

In this prospective observational study, we used a range of quantitative data collection approaches to accomplish the study aims. We used the RE-AIM framework to investigate the impact of the checklist. Reach is an individual-level measure of the participation, and will be investigated by the proportion of eldercare workers who know the checklist and the characteristics of those who know the checklist compared to a reference group (union members who didn't answer, that they have gained knowledge of the checklist during the campaign (answered no or don't know). In addition to this, reach will be measured as number of unique visitors to the online checklist website (day to day activity, accumulated activity, and geographical position) during the campaign. Effectiveness will be evaluated in respect to whether the prioritization of the work environment among workplaces that know the checklist has changed. Adoption is an organizational-level measure of the representativeness of the setting, and will be investigated by the proportion of workplaces who adopt the checklist (create an account and log into the online checklist website) and their characteristics. Implementation refers to the extent to which an intervention is delivered as intended (dissemination) and will be measured at individual level as whether the eldercare workers has seen a diploma at their workplace. At the organizational level, implementation will be measured as workplace activity at the online checklist website. Maintenance is the extent to which the programme becomes a part of the routine at the workplace and will be evaluated by return/revised actions (repeated use of the checklist). See protocol paper for further details (18).

### Data Collection and Outcomes

**Figure 1:**
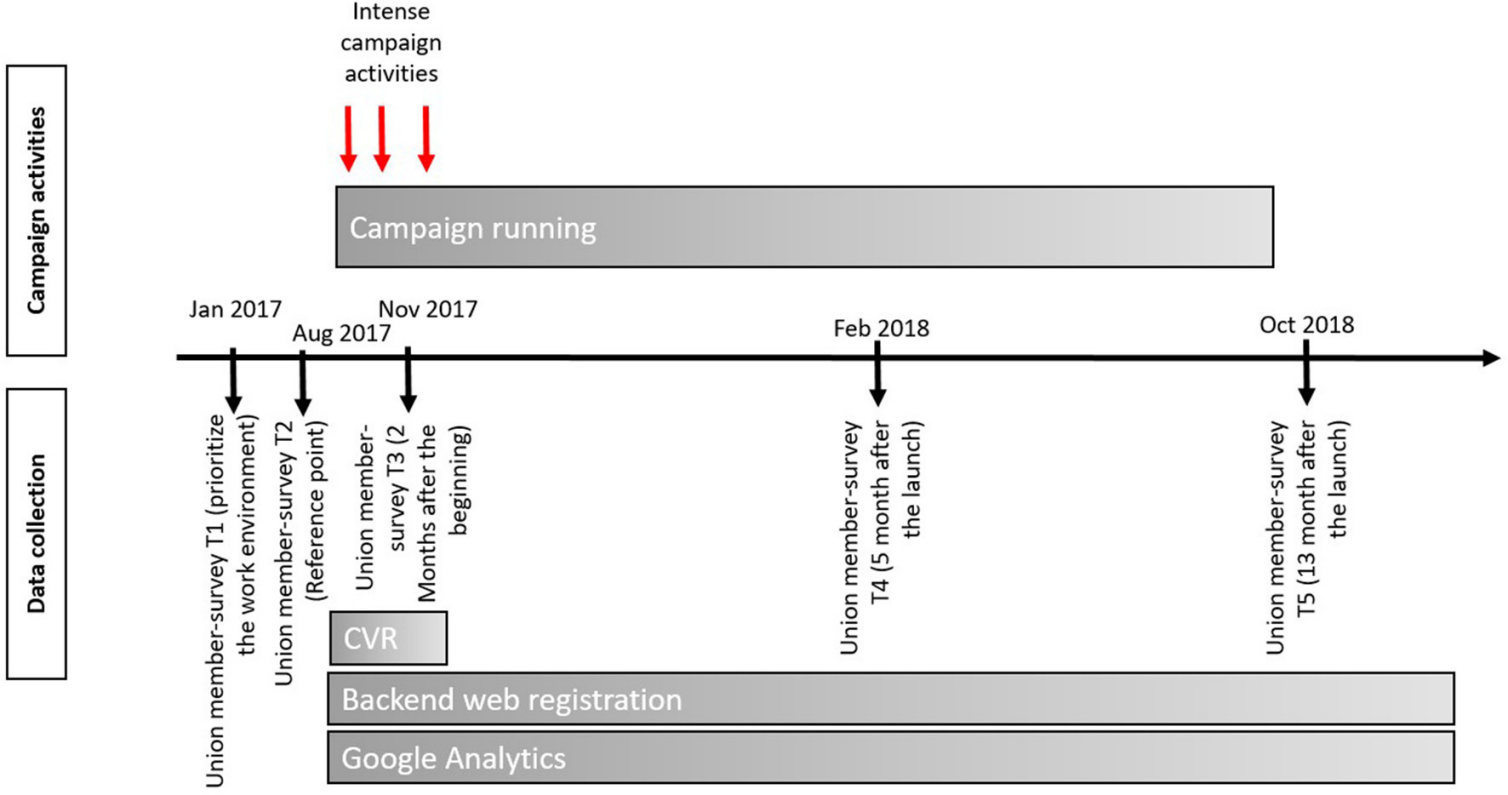
Overview of the campaign activities and the data collection during the campaign period.

We used multiple data sources to report reach, effectiveness, adoption, implementation and maintenance. The data sources included data from a large union survey, the checklist website, the Central business Register (CVR—contains information about all registered workplaces in Denmark) and Google Analytics. See Figure 1 for detailed data collection points before, during and after the campaign.

#### Union Survey

The third largest trade union in Denmark (FOA), organize ~180,000 members primarily in the public sector. Members can voluntarily sign-up to receive a questionnaire 4–6 times a year. Union members can register and drop out as they want, making the population an open cohort. The union survey is sent to ~7,500 union members each time. For each round of the survey, we embedded campaign-specific questions in the questionnaire and the following background information on each member was collected: age, gender, position of trust (OHS representative, employee representative, OHS representative and employee representative or no position of trust) and manager (yes/no), information on employer (municipality or an self-governing institution, private/private resident, region, state, or other/don't know) and workplace (temporary agency, treatment/district psychiatry, home care, social psychiatry, school, rehabilitation, hospital, nursing home, special area, handicap assistant, or other).

*Reach* was assessed with the questions: “do you know the campaign (MEDvirk)? (Yes, no or don't know),” “where have you heard of the campaign (MEDvirk)? (network, OHS representative, colleagues, the Danish Working Environment Authority, employer/sector association, the Sector-Specific Work Environment Community Organization for Public and Welfare workplaces, trade union (FOA), website/newsletter, conference or similar, flyer, other or don't know/don't remember) (you can answer more than one)).” The union members were invited to answer these questions just after the launching of the campaign (T3: 17th to 28th of November 2017), 5 months after the launching (T4: 2th to 14th of February) and after the campaign finished (T5: 12th to 31th of October 2018). Further before the campaign started, it would have been impossible to know the checklist and to establish a reference point we invited the union members before the campaign started (T2: 21th to 31th of August 2017) to answer the same questions as a control.

*Implementation* was assessed with the question: “have you seen this diploma at your workplace? (yes, no or don't know)” (showing a picture of the diploma). The union members were then invited to answer the question just after the launching of the campaign (T3: 17th to 28th of November 2017), 5 months after the launching (T4: 2th to 14th of February) and after the campaign finished (T5: 12th to 31th of October 2018). Again as a control, the union members were invited to answer the same question before the campaign started (T2: 21th to 31th of August 2017).

Furthermore, in the last union survey and in an additional union survey from January 2017 (T1) we included a question to evaluate *effectiveness*: “does your workplace in general prioritize the work environment? (to a very great extent, to a great extent, to some extent, to a small extent, not at all or don't know).” See protocol paper for overview of the data collection and timeline (18).

#### The Checklist Website

From the checklist website, we used user-specific information from each visitor and work environment action. User data were copied from “www.MEDvirknu.dk” user database and pasted into an “.xlsx” file. Copied Metrics were “user name,” “municipality,” “workplace name,” and “unique company identification (p-Number).” All actions were downloaded as an “.xlsx” file (incorporated function in the backend of the website), including “municipality,” “workplace name,” “unique company identification (p-Number),” “email,” “unique user-id,” “date for created action,” “number of visits,” “number of actions,” “action,” “date for edit,” “print,” and “answers to the 11 checkpoints.”

To assess *adoption and maintenance* both dataset were filtered to only include users/actions from 4th of September 2017 until and including the 3rd of September 2018. Additionally, data were filtered via user names and/or email to remove project developer, internal and project associate users created to highlight the checklist to potential users. The unique company identification (p-number) at the user-specific dataset was linked to information from the CVR to evaluate the representativeness and characteristics of adopters. The CVR dataset from “www.datacvr.virk.dk/data/” was filtered to include all possible eldercare workplaces in Denmark (including hospitals, home care, nursing home, 24-h care center (mental- or physical disability or children and adolescents) or others based on main and secondary sector (4,899 different eldercare workplaces in Denmark). The information contained in the CVR is: size (number of employees in intervals [ <49 (small) 50–199 (medium) >200 (large)]), type of workplaces (nursing home, home care, hospital, etc.), age (date for founding of the workplace), and geographical position of the workplaces [municipalities (region)].

To assess *implementation*, we coded actions into the following different categories: physical surroundings, physical exposure, psychological exposure, training during working hours, organization, other or not usable.

#### Google Analytics

The website “www.MEDvirknu.dk” was associated with a Google Analytics account. We downloaded day-by-day history as “.xlsx” files from this account, between and including 4th of September 2017 and 3rd of September 2018. The metrics downloaded were “date,” “segment,” “users,” and “bounce rate.” “Users” indicate unique visitors, and will be used as a measure for reach. Furthermore, month-by-month geographical data on city level was also downloaded as “.xlsx” files from the google analytics account, from and including 4th of September 2017 and 31st of August 2018. Google Analytics determine geographical location by the active users' IP-address. The metrics downloaded were “date-interval,” “segment,” “users,” “city,” “month,” and “year.”

User segment for both data downloads were limited to users from the geographical location of Denmark. Month by month geographical data were linked to a dataset from Statistics Denmark including all cities in Denmark with more than 200 citizens and the belonging municipality (98 municipalities in Denmark).

The activity at the checklist website was used to evaluate *reach* (number of unique visitors to the online checklist website (day to day activity, accumulated activity, and geographical position) during the campaign) and the activity after different dissemination activities.

### Analyses

To test for differences between the workplaces that use the checklist (adopters) and workplaces that do not use the checklist (non-adopters) we performed ANOVA and *t*-test. To test for differences between the union members who know the checklist (reached) and union members who do not know the checklist (non-reached) we performed ANOVA and *t*-test.

Union members who answered that they knew the campaign or had seen the diploma before the start were excluded in the analysis. For the analysis of where the members have heard of the campaign, each time a union member has participated in the survey and knows the campaign, the answer is included in the analysis (minimum once and maximal three times).

For the effectiveness evaluation the inclusion criteria were: participation in T1 and T5. We excluded respondents who answered “don't know” and respondents who knew the campaign before campaign start (T2). Respondents who knew the campaign was based on T3, T4, and T5. To investigate the prioritizing of work environment before the campaign started and after the campaign, we performed a repeated ANCOVA with time (T1 and T5) as the within-participants factor, and the between-participants factor (knows the campaign) as the dependent variable. We adjusted for age and gender.

## Results

### Dissemination

Table 1 provides an overview of the dissemination activities to promote the checklist over a period of 12 months. The dissemination activities included digital elements, physical appearance as e.g., oral presentations, campaign materials, and printed elements. The checklist and all the activities were in Danish. Approximately 55,000 people were subscribed to receive the newsletters by the different partner organizations where the checklist was promoted and more than 500 people participated in oral presentations or training sessions (physical presence). There were more than 26,000 likes on partner organizations' Facebook who shared the checklist, the campaign video or articles about the checklist. Paper elements (a letter, printed checklist, and magnets) were sent to 1,079 nursing homes, 627 home care units, and 98 different administrative departments of the municipalities.

**Table 1:** Dissemination activities to promote the checklist (MEDvirknu.dk) during the 1-year campaign.

**Timing**	**Dissemination Action**	**Reach**		**Adopting**			
		**Potential**		**Eligible workplaces**		**Ineligible workplaces**	
	**Digital elements**	**Reached/n^**d**^**	**Reached/n^**e**^**	**Workplaces/n^**e**^**	**Actions/n^**e**^**	**Workplaces/n^**e**^**	**Actions/n^**e**^**
04-Sep-17	Checklist shared on the project group's own LinkedIn profiles	NA	111 (298)	0 (7)	0 (9)	4 (10)	4 (11)
	Article about the checklist on the Sector-Specific Work Environment Community Organization for Public and Welfare workplaces ()	NA					
	News about the checklist on the Danish Knowledge Center for Work Environments webpage	NA					
	Special newsletter about the checklist to those subscribed to receive the Danish Knowledge Center for Work Environment's newsletter	21,000 subscribed^a^					
	News about the checklist shared on the project leader's (researcher) LinkedIn profile (Danish and English)	669 followers^b^					
05-Sep-17	Checklist shared on The Danish Working Environment Authorities' LinkedIn	4,189 followers^b^	187 (341)	7 (12)	9 (16)	6 (39)	7 (41)
	Online article in the sector-based magazine “Pleje” (care) about the checklist	NA					
	*News about the checklist at the National Research Center for the Working Environment's official website (www.nfa.dk)*	NA					
07-Sep-17	Newsletter about the checklist to those subscribed to receive The National Research Center for the Working Environment's newsletter	4,100 subscribed^a^	92 (132)	1 (2)	1 (2)	5 (8)	5 (8)
	News about the checklist on a Facebook page generated in the development process of the checklist, primarily targeted at workers in the eldercare sector with the aim of sharing knowledge on how to create a good work environment (The page is named “SKAB JER”)	253 Facebook likes^b^					
14-Sep-17	Checklist shared on “FOA Vejle's” Facebook (sub-group within the large union within the sector)	411 Facebook-likes^b^	36 (80)	1 (1)	1 (1)	2 (7)	3 (8)
15-Sep-17	Checklist shared on The National Research Center for Working Environment's Facebook page	1,448 Facebook likes^c^	44 (49)	0 (0)	0 (0)	5	5 (5)
27-Sep-17	Newsletter about the checklist to those subscribed to receive the Sector-Specific Work Environment Community Organization for Public and Welfare workplaces	9,876 subscribed^a^	153 (237)	3 (7)	3 (7)	3 (4)	3 (5)
	Checklist shared on “Godt arbejdsmiljø”s (good working environment) Facebook group (associated with the Sector-Specific Work Environment Community Organization for Public and Welfare workplaces)	16,888 Facebook-likes^b^					
28-Sep-17	Newsletter about the checklist to those subscribed to receive The Danish Working Environment Authorities' newsletter	20,000 subscribed^a^	84 (108)	4 (4)	4 (4)	1 (1)	2 (2)
	Repost of “Godt Arbejdsmiljø”s Facebook post, on “SKAB JER”s Facebook	253 Facebook-likes^b^	24 (36)				
10-Oct-17	Campaign movie about the checklist shared on “SKAB JER” Facebook page	253 Facebook-likes^b^/4,400 views of the campaign movie^c^	23 (54)	0 (0)	0 (1)	0 (0)	0 (0)
10-Oct-17	Campaign movie about the checklist shared on The National Research Center for Working environments' Facebook page	1,448 Facebook-likes^c^/4,400 views of the campaign movie^c^					
19-Oct-17	Checklist shared on “SKAB JER”s Facebook page	253 Facebook-likes^b^	12 (38)	0 (0)	0 (0)	0 (1)	0 (1)
19-Oct-17	Campaign movie about the checklist shared on The Danish schools for nursing aides' Facebook	2,793 Facebook-likes^b^					
24-Oct-17	Campaign movie about the checklist shared on “Godt arbejdmsiljø”s (good working environment) Facebook	16,888 Facebook-likes^b^	31 (47)	0 (0)	0 (0)	0 (1)	0 (1)
14-Nov-17	Newsletter about the checklist to those subscribed to receive the Sector-Specific Work Environment Community Organization for Public and Welfare workplaces newsletter	9,876 subscribed^a^	88 (123)	1 (1)	1 (1)	3 (3)	3 (3)
07-Dec-17	Newsletter about the checklist to those subscribed to receive Danish physiotherapists' newsletter	NA	43 (65)	0 (0)	0 (0)	0 (0)	0 (0)
	Internal newsletter about the checklist to employees in the Ministry of Employment	NA					
15-Jan-18	Article about the checklist shared on topic-specific Facebook page regarding interventions for musculoskeletal health at public workplaces, published and edited by a section under the Ministry of Employment	18,477 Facebook-likes^b^	11 (20)	0 (0)	0 (0)	0 (0)	0 (0)
30-Jan-18	Repost of the Sector-Specific Work Environment Community Organization for Public and Welfare workplaces article on “SKAB JER”s Facebook	253 Facebook-likes^b^	42 (72)	0 (0)	0 (0)	2 (2)	3 (3)
30-Jan-18	News at the Sector-Specific Work Environment Community Organization for Public and Welfare workplaces website (www.arbejdsmiljoweb.dk) - interview with the project leader	NA					
14 May-18	Checklist shared on “Godt arbejdsmiljø”s (good working environment) Facebook	16,888 Facebook-likes^b^	28 (87)	0	0 (0)	0 (1)	0 (0)
15 May-18	Checklist shared on “Godt arbejdsmiljøs” (good working environment) Facebook	16,888 Facebook-likes^b^	59 (73)	1 (1)	1 (1)	1 (1)	1 (1)
	**Physical presence**	Participants/*n*	Reached/n^f^	Workplaces/n^f^	Actions/n^f^	Workplaces/n^f^	Actions/n^f^
05-Sep-17	Oral presentation of the checklist at the yearly Working Environmental Conference organized by the Local Government Denmark (central organization of all Danish municipalities), with representatives from most of the Danish municipalities	≈100 participants	187	7	9	6	7
06-Sep-17	Oral presentation at a conference for local work environment representatives within the eldercare sector—organized by the sector-specific work environment Community Organization for Public and Welfare workplaces	≈250 participants	154	5	7	33	34
	Training in use of the website and checklist of all Danish Working Environment Authority inspectors within the elder care sector	≈90–100 participants					
21-Sep-17	Oral presentation of the checklist at the trade union for eldercare workers—FOA—for employee representatives situated at regional union offices (i.e., coordinators and advisors of local employee representatives at workplaces)	NA	40	6	6	4	4
5-Oct-17	Oral presentation for the Working Environment Authorities employee club of therapists (typically inspectors) (project leader promoting the checklist)	≈40 participants	46	6	6	13	13
25-Oct-17	Oral presentation at the yearly conference for teachers in the common labor parties' school for work environment that trains work environment representatives in Denmark.	NA	16	0	0	1	1
26-Oct-17	Oral presentation at the yearly conference for teachers in the common labor parties' school for work environment that trains work environment representatives in Denmark.	NA	11	0	0	0	0
22-Nov-17	Theme-day for work environment groups in eldercare sections in a municipality	≈80 participants	53	13	14	8	7
28-Nov-17	Oral presentation at the work environment conference for work environment consultants and other occupational health and safety representatives (not sector-specific)	52 participants	47	0	0	2	2
22-Jan-18	Instruction and facilitation of usage of the checklist for work environment groups in a municipality	13 participants	14	3	7	1	1
22-Mar-18	Network meeting for OSH representative (not sector-specific)	≈40 Participants	40	6	6	23	26
	**Articles in magazines**	Circulation/*n*					
5-Sep-17	Article about the checklist in the magazine “Arbejdsmiljø” (Working Environment), which covers working environment and is distributed by a section under the Ministry of Employment	6,600					
29-Sep-17	Article about the checklist in the magazine “Pleje” (Care)	7,944					
17-Apr-18	Article about the checklist in the magazine “Arbejdsmiljø” (Working Environment)	6,600					
	**Campaign materials**	Views/*n*					
10 Oct-17	Campaign movie published	717 views^b^					
9 Oct-17	Introduction movie of the checklist published	695 views^b^					
	**Paper elements**	Sent out/*n*					
Dec-17	Letter, 5 × postcards and one magnet	1,079 nursing homes and 627 home care					
	Letter and 3 × postcard	98 different administrative departments of the municipalities					

*Data collected per: ^a^5 Apr 2017, ^b^18 Dec 2018, and ^c^3 Jan 2019 NA, not available ^d^Number of followers/subscribed and ^e^Number of reached/adopting workplaces/actions the day, the digital element occurred (Number of reached/adopting workplaces/actions the day, the activity occurred + the day after the digital element occurred), ^f^Number of reached/adopting workplaces/actions the day, the dissemination activity occurred*.

*For digital elements, you can see the reach and adopting workplaces and actions for the day of the activity and the day after. For other dissemination elements, you can see the reach and adopting workplaces and actions*.

In Table 1, the number of potential reached and the actual reached can be seen. Within each activity, we estimated the potential reach. For the digital elements potential reach included likes, followers and subscribers. For the physical presence potential, reach included participants. In addition, the number of adopting workplaces can be seen from the different dissemination methods used to promote the checklist. This provides an overview of the effect of the different dissemination strategies. Table 1 shows that oral presentations and meetings may be good strategies to reach people. Several times we reached a high proportion of the potential reached during oral presentations and training sessions (physical presence) compared to dissemination actions using digital elements, As an example, the 6th of September we held an oral presentation for around 250 participants (the same day we also had a training session for around 90–100 participants), resulting in 154 visits. The 27th of September we shared the checklist on Facebook with a potential reach of around 17,000 and a newsletter was sent to almost 10,000, resulting in 153 (237) visits.

In Table 2, an overview of where the respondents have heard of the campaign during the 1-year campaign is presented. During the campaign, 1,168 respondents answered the question (response rate 99.7% of all who indicated to know the checklist). Almost 60% have heard of the campaign from the trade union FOA, more than 30% from their OHS representative and around 20% have heard of it from colleagues or network.

**Table 2:** Overview of where the survey-members have heard of the campaign.

**Knowledge of the checklist (*****N*** **=** **1.168)**		
	***N***	***%***
Trade union (FOA)	681	58
OHS representative	400	34
Network	233	20
Colleagues	214	18
Website/newsletter	196	17
Brochure/flyer	83	7
Don't know/remember	79	7
The Sector-Specific Work Environment Community Organization for Public and Welfare workplaces	73	6
The Danish Working Environment Authority	47	4
Employer/sector association	46	4
Conference	31	3
Other	30	3
Answers	2.113	–

*OHS, Occupational Health and Safety*.

### Reach (Organizational Level)

#### Unique Visitors

**Figure 2:**
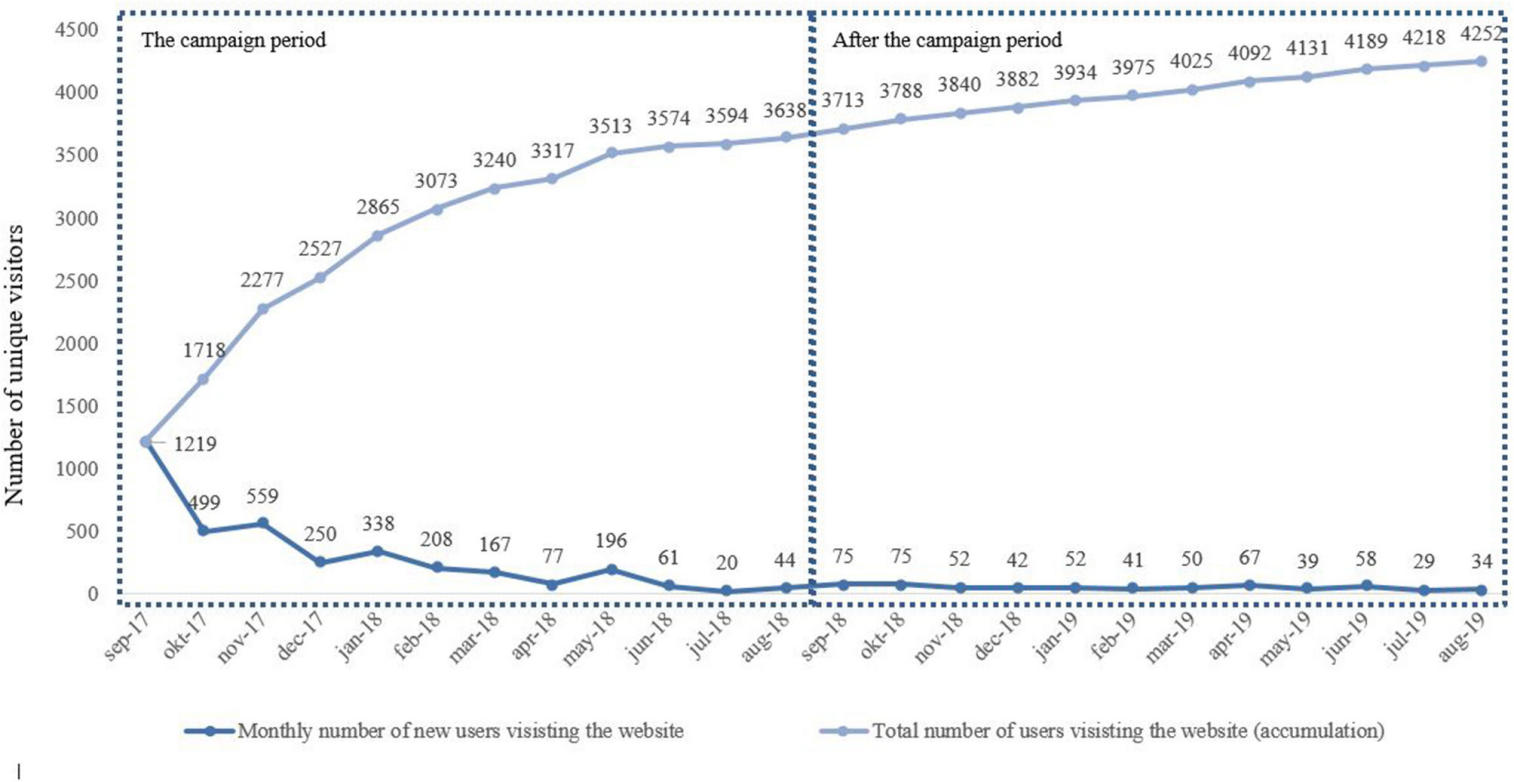
Overview of the accumulated and monthly Danish visitors during the campaign period and after the campaign period.

From 4th of September 2017 until 3rd of September 2018, we had 3,644 unique visitors to the website hosting the digital checklist (average of 10 unique visitors per day during the 364 campaign days). Within the first 3 months, we had 2,277 unique visitors to the website (average of 25 unique visitors per day during the first 3 months). The number of unique visitors peaked within the first couple of months; within the last 6 months of the campaign, 571 unique visitors visited the website (average of 3 unique visitors per day during the last 6 months). The bounce percentage, i.e., percentage of visitors leaving the website after only viewing one page, was 30% (*n* = 1,089). After the campaign period and until September 2019 people still visited the website, however the unique number of visitors decreased after the campaign period (average of 2 unique visitors per day after the campaign). Figure 2 gives an overview of the monthly and total number of visitors on the website during and after the campaign.

#### Geographic Location

Within the 1st month of the campaign, visitors from 82 different municipalities in Denmark were reached (84%). Within the same period, 39 municipalities were reached with at least five unique visitors from each of the 39 municipalities. This number was unchanged for the rest of the campaign period. After 3 months, 89 different municipalities in Denmark were reached (91%). After 1 year, visitors from 94 different municipalities in Denmark were reached (96%) (data not shown).

### Adoption

In total, 534 visitors (*n* = 15% of unique visitors) created an account and logged in to the website (adopters). Within the 12 months of the campaign, visitors from 88 different municipalities created an account and logged in to the website (from 56 visitors we had no information about municipality). Top three adopting municipalities were all placed in Zealand (10% of all eligible workplaces were located in Copenhagen, 8% in Frederiksberg, and 7% in Ringsted).

**Figure 3:**
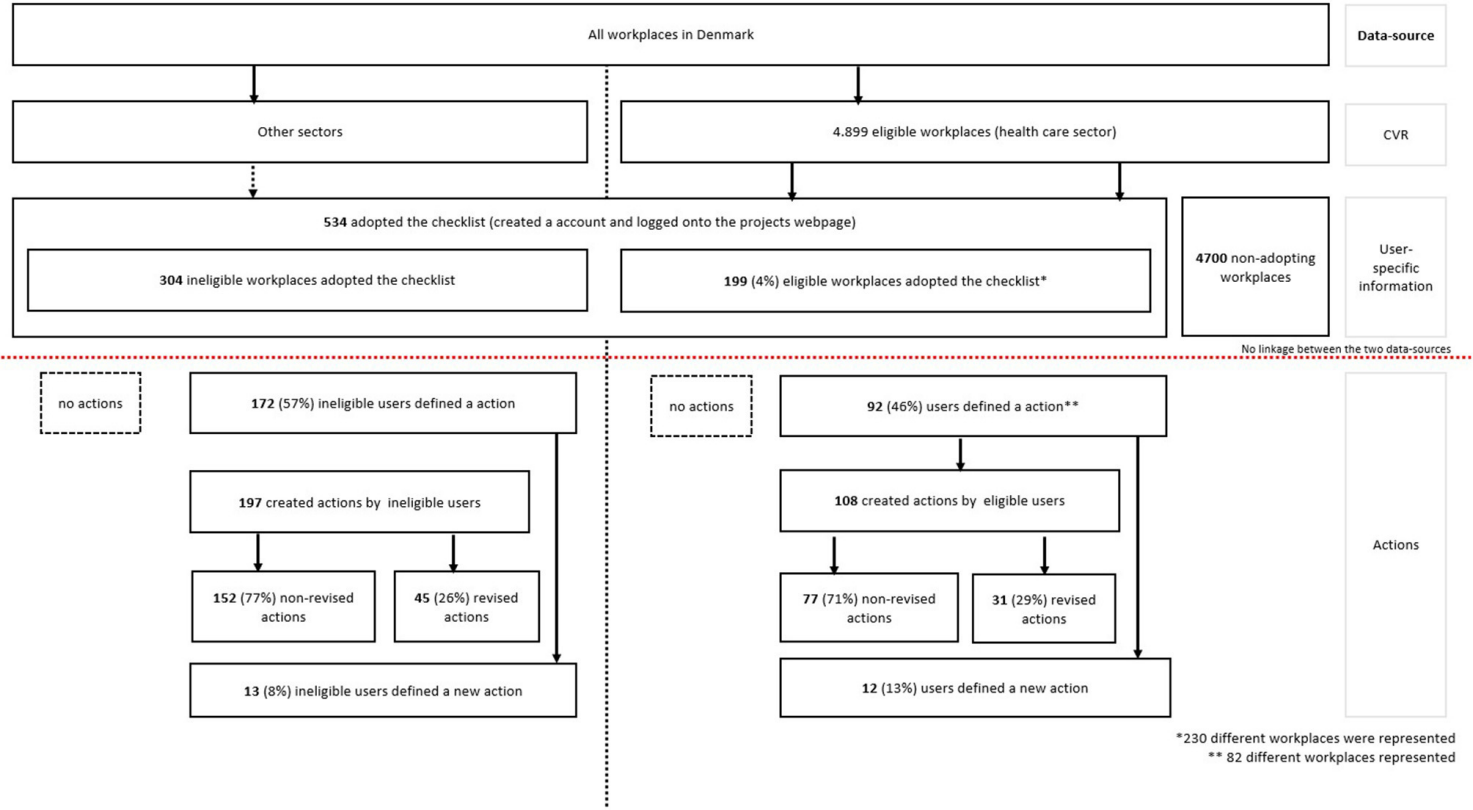
Flow diagram showing adopters and users of the checklist among eligible/ineligible workplaces in Denmark.

Of the 534 created accounts, 230 of the accounts were linked to a target p-number, from 199 different workplaces (from 1 to 7 accounts per workplace) and defined as eligible workplaces, meaning that 4% of all possible eldercare workplaces in Denmark adopted the checklist. A special focus within the dissemination strategy was the nursing homes. Of 1,089 nursing homes, 88 nursing homes adopted the checklist, corresponding to 8%. Of the 199 identified workplaces, the users came from 74 different municipalities within Denmark (data not shown). There were 304 of the created accounts that were without a p-number, and defined as ineligible workplaces (from other sectors than the eldercare sector). Some of the adopting ineligible workplaces were from unidentified eldercare workplaces, however a large amount were from other sectors (schools, administrations, municipality, childcare, the Danish Working Environment Authority, and trade union). See Figure 3, for a flow diagram of possible adopters, adopters, and use of the checklist.

In Table 3, characteristics of the workplaces that adopted the checklist and workplaces that did not adopt the checklist are presented. A significant higher proportion of workplaces that adopted the checklist were placed in the capital region, were more often nursing home, home care, or hospital, medium sized workplaces or founded before year 2000 compared to workplaces that did not adopt the checklist.

**Table 3:** Characteristics of the workplaces who adopted and did not adopt the checklist.

**Characteristic of the workplace**	**Adopting workplaces (*****N*** **=** **199)**		**Non-adopting workplaces (*****N*** **=** **4,700)**		**Differences adopting/non-adopting workplaces**
**Geographical position (regions)**	***N***	**%**	***N***	**%**	***p*-value**
North Jutland	22	11	684	15	0.011
Central Jutland	40	20	1,060	23	
The Southern part of Denmark	44	22	1,058	23	
Zealand	30	15	849	18	
Capital	63	32	1,049	22	
**Type of workplace (based on main sector)**					0.000
Nursing home	88	44	1,001	22	
Home care	33	17	571	12	
Hospital	21	11	242	5	
24-h care center (mental disability)	22	11	1,048	23	
24-h care center (physical disability)	9	5	285	6	
24-h care center (children and young)	1	1	453	10	
Other	25	13	1,100	24	
Size of workplace (Employees)*					0.000
Small	62	35	2,257	65	
Medium	91	51	1,127	32	
Large	24	14	104	3	
Missing	22	–	1,212	–	
Size of workplace (fulltime employees)*					0.000
Small	83	47	2,610	75	
Medium	73	41	802	23	
Large	21	12	76	2	
Missing	22	–	1,212	–	
**Workplace start-up**					0.000
Before 2000	101	51	1,584	34	
2000–2010	44	22	1,191	25	
After 2010	54	27	1,925	41	
*in 2015					

### Reach—Individual Level (Knowledge of the Campaign)

Seven thousand three hundred and fifty-four union members from the Social and Health Service Sector were invited to participate in the survey before the campaign started−2,574 answered (response rate 35%). There were 181 survey members who responded that they knew the checklist before the campaign started (143 union members participated in the following three rounds and were excluded) and 152 had seen the diploma before the campaign started (123 union members participated in the following three rounds). Two months after the launch of the campaign 7,917 union members were invited to participate in the survey−2,891 answered (response rate 37%). Five months after the launch, 7,875 union members were again invited−2,877 answered (respond rate 37%). Finally, after the campaign finished (13 months after the launch), 8,399 union members were invited−3,055 answered (respond rate 36%). before, during and after the campaign, 5,118 unique union members participated in the survey. During and after the campaign, 4,692 unique union members answered the question. of these, 17% (*n* = 754), of the union members answered at least once, that they have gained knowledge of the checklist during the campaign and were considered reached. However, given that the total number of eldercare workers are ~100,000, the reach of total eldercare workers in Denmark can be considered to be 0.8%. Two months after the launch of the campaign, 9% (*n* = 252) of the union members were reached. Five months after the launch, 13% (*n* = 367) of the union members were reached and after 13 months (after the campaign finished), 13% (*n* = 380) of the union members were reached.

In Table 4, characteristics of reached and non-reached union members are presented (characteristics from the first time the respondent was reached). Overall, the characteristics are similar. However, a higher (non-significant) proportion of the reached had a position of trust than the non-reached.

**Table 4:** Characteristic of the Union (FOA) survey-members who were “reached” before the campaign started (and therefor excluded), reached during the campaign and not reached.

**Characteristic of FOAs survey-members**	**Reached before the campaign started (*****N*** **=** **181)**		**Reached (*****N*** **=** **754)**		**Non-reached (*****N*** **=** **3.795)**		**Differences between reached/non reached**
	***N***	**%**	***N***	**%**	***N***	**%**	***p*-value**
Gender (women)	164	91	691	92	3.466	91	0.779
Age (years (SD years))	52.5 (9.0)		50.1 (10.2)		49.7 (10.3)		0.461
Manager (yes)	7	4	6	1	54	1	0.168
**Position of trust**							0.070
No position of trust	113	62	512	68	3,272	86	
Employee representative	43	24	121	16	318	8	
OHS representative	24	13	117	16	187	5	
OHS representative and employee representative	1	1	4	1	18	0	
**Employer**							0.116
Municipality or an self-governing institution	142	78	627	83	3.082	81	
Region	29	16	93	12	530	14	
Private/private resident	7	4	26	3	151	4	
State	1	1	0	0	5	0	
Other/don't know	2	1	7	1	24	1	
Missing	–	–	2	–	3	–	
**Workplace**							0.885
Nursing home	72	40	321	43	1,591	42	
Home care	48	27	210	28	1,083	29	
Hospital	22	12	58	8	339	9	
Other	39	22	165	22	782	21	

*OHS, Occupational Health and Safety*.

*The number and percent or mean and standard deviation (SD) are presented*.

### Implementation

#### Organizational Level

Of the 199 eligible workplaces (in total 230 accounts, meaning some workplaces created more than one account), that created an account on the website, 92 workplaces (46%) defined an action and 12 (13%) workplaces defined a second action. Overall 108 different actions were defined. However, a higher proportion of the users who created an account from ineligible workplaces defined an action, compared to users from eligible workplaces. Table 5 shows the types of actions defined by eligible workplaces. The most common action was related to improvements in the physical surroundings and the least common action was change in the physical exposures.

**Table 5:** Categories of actions and revised actions defined by eligible workplaces, listed according to frequency (high-low).

**Action**	**Actions by eligible workplaces**		**Revised actions by eligible workplaces**	
	***N***	**%**	***N***	**%**
Physical surroundings (e.g., use of assistive devices/reduce noise)	25	23	7	23
Physical exposure (e.g., reduce lift/awareness of pain)	11	10	5	16
Psychological exposure (e.g., reduce workload/no bullying)	14	13	6	19
Training during working hours (e.g., cardio/elastic training)	15	14	4	13
Organization (e.g., meeting/communication/collaboration/education/information)	20	19	4	13
Other (e.g., improve the work environment/starting different processes)	17	16	4	13
Not usable	6	6	1	3
Total	108	100	31	100

**Figure 4:**
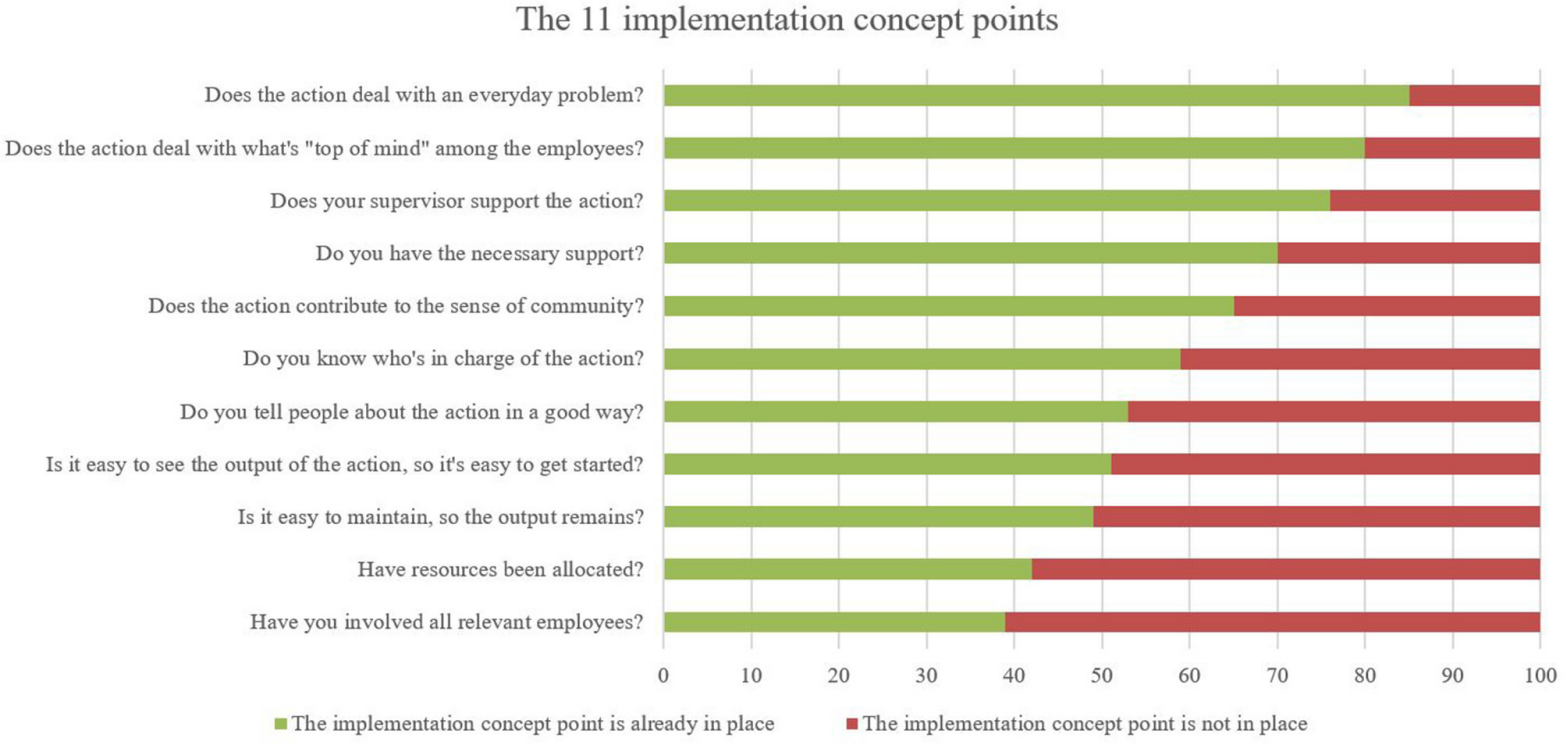
The distribution of workplaces where the implementation concept point was already in place or not in place by the time they filled in the checklist by eligible workplaces (high to low).

Figure 4 shows how frequent workplaces checked that the implementation concept point was already in place or not, by the time the workplaces filled in the checklist. The three implementation concept points which were most frequently already in place by the time the workplaces filled in the checklist were “*does the supervisor support the action*?,” “*does the action deal with what's* “*top of mind*” *among the employees*?” and “d*oes the action deal with an everyday problem*?” The two implementation concept points, which were most frequently not in place, was “h*ave you involved all relevant employees?*” and “h*ave resources been allocated?*”

#### Individual Level

Table 6 shows the distribution of printed diplomas, letters and tips by non-revised and revised actions. A higher proportion of users who revised their action printed the diploma, letter and tips, compared to the non-revised actions. During and after the campaign, 4,569 unique union members answered the question whether they had seen the diploma at their workplace. Of these, 17% (*N* = 762) answered at least once, that they had seen the diploma at their workplace. Two months after the campaign started 8% (*N* = 210) had seen the diploma at their workplace, 5 months after the campaign started, 15% (*N* = 429) had seen the diploma at their workplace and after the campaign finished, 12% (*N* = 343) had seen the diploma at their workplace.

**Table 6:** Distribution of printed diploma, letter and tips by non-revised and revised actions by eligible workplaces.

	**Non-revised actions (*****n*** **=** **77)**		**Revised actions (*****n*** **=** **31)**	
	**N**	**%**	***N***	**%**
**Print**				
Diploma	36	47	26	84
Letter	15	19	12	39
Tips	29	38	15	48

### Maintenance

There were 31 (29%) of the actions that were revised (see Figure 3). Of the workplaces who defined an action, a higher proportion of the users who were employed at eligible workplaces returned to the website and defined a new action, compared to users from ineligible workplaces. From eligible workplaces a higher proportion of the users revised their action. For eligible workplaces who revised their action, 35% revised the action the same day, 20% revised their action within 1 week, 16% revised their action within 30 days (and more than 7 days), and the remaining 29% revised after 30 days (maximum 202 days after the action were created). Table 5 shows revised actions defined by eligible workplaces.

### Effectiveness

The unadjusted and adjusted (for gender and age) mean rating of the prioritization of the work environment for reached and non-reached union members, before the campaign started (baseline) and after the campaign, remained the same (0.35) and there was no significant group by time effect. However, there was a significant difference between the reached and non-reached union members. In general, reached union members were employed at workplaces, where the prevention of the work environment were prioritized to a higher degree, compared to non-reached union members (<0.001).

## Discussion

We evaluated the dissemination of a checklist for improving implementation of work environment initiatives in the Danish eldercare using the RE-AIM framework. One year after the launch of the campaign, almost all municipalities in Denmark had visited the website (96%). Among all eligible workplaces, 4% of eligible workplaces actually adopted the checklist covering 8% of all nursing homes in Denmark. In the following, we will discuss whether this is a satisfying number of adopters, what affected the adoption percentage, and whether adopters succeeded with implementation.

### The Impact of Different Dissemination Strategies

Due to several activities at the same time, it is not possible to isolate the impact of each activity separately. However, one of our main findings regarding dissemination of the checklist was that physical presence at workplaces, at sector-specific conferences and at meetings was an effective way to reach the target population. This is emphasized in our data, as the three municipalities adopting the checklist the most, are also the municipalities we visited the most during the campaign. Andersen and colleagues found that workplace visits affected the number of website visits that are in line with our findings (21). The strategy was that the OHS representatives and employee representatives at the workplaces would disseminate the knowledge about the checklist further to the rest of the employees in their respective workplaces (22). Our findings show, that we succeeded in doing this, as the trade union FOA and OHS representatives are the most frequent sources from which the respondents have heard of the checklist. Further, network and colleagues were the third and fourth highest ranked sources. This is in accordance with previous findings that the channels that intended audiences already trust and access for information is crucial (10). It seems like the dissemination strategy was successful in terms of covering Denmark geographically. A year after the campaign started, we succeeded in reaching 96% of the municipalities in Denmark.

### Reached Eldercare Workers

The campaign reached 17% of the respondents from the union survey—this corresponds to a reach of 17,000 eldercare workers nationwide, if assuming that the survey is representative. Another national campaign in Denmark targeting public-sector employees with a mixture of networking activities, workplace visits, and a mass media outreach with topics related to job and body (e.g., musculoskeletal pain, movement and work), also reached 17% of their target population over a period of 3 years (21). Although previous findings also suggests that campaigns using internet and social media seem to reach workplace-based audiences, how to best reach the employees who are audiences for OHS information remains a challenge (23, 24).

Among the sample of reached employees, employees with a position of trust (OHS or employee representatives) were over-represented. Further, the reached employees were employed at workplaces where they reported the work environment to be more highly prioritized than non-reached employees reported. A highly prioritized work environment effort may indicate good organization around the work environment practice. The checklist is highly adaptable to fit in line with a well-organized and structural work environment practice, and this may explain our reach of employees from workplaces with higher prioritization. For example, for the checklist to be useful, the workplaces should be quite aware what actions they aim to implement and why. Furthermore, previous studies have shown that workplace readiness for change is a strong indicator for a successful implementation of workplace health programs (25). A good work environment practice may also cultivate the readiness for change. To target workplaces where the work environment is less prioritized may require a special focus on preparation and creating organizational readiness to work with work environment issues and a special focus in the dissemination strategy.

### Which Workplaces Use the Checklist and How?

The campaign disseminated the checklist to all regions in Denmark, and 199 workplaces adopted the checklist, corresponding to 4% of all eligible workplaces in Denmark. A special focus within the dissemination strategy was the nursing homes. Of 1,089 nursing homes, 88 nursing homes adopted the checklist, and corresponding to 8%.

While 4% is a low fraction, we consider 199 workplaces and 8% of all nursing homes in Denmark in action after a campaign quite a success. If all 199 workplaces get an actual output of the checklist, this is likely to affect ~4,000 employees' work environment positively. Considering the fact that the campaign was merely motivating—with no incitements or legal requirements, and the fact that it was a relatively low financed campaign merely financed by research funding, the dissemination and workplace adoption rate was successful. It is even likely, that having continued the intense campaign of the first 3 months for a longer period, and supporting this with more good examples of implementation, could have increased dissemination further. However, the biggest issue regarding gaining impact of this campaign does not seem to be the dissemination—but in the implementation and maintenance.

Developing both the content and the concept of the checklist with much user and stakeholder involvement, higher implementation rates than 46% of the adopters could likely have been expected. Furthermore, a relatively low number of the adopters (13%) used the checklist twice, indicating, that the incentives for returning to the checklist were not strong enough. However, we have data showing that the use of the checklist was broad in scopes [both physical and psychosocial work environment challenges were addressed (see Table 5)] and the workplaces generally reported to be lacking several of the implementation concept points to reach full implementation, both of which may explain the relatively low implementation rate. Regarding the broadness in scope,—this is in line with the aim of the checklist a possible explanation for this is the complexity of multiple work demands resulting in many different work environment challenges. Rasmussen et al. (26) find a similar trend among the same target population. Regarding the implementation concept points lacking among the users, most actions were supported by the supervisor, in line with what was “top on mind” among employees and dealt with an everyday problem (27, 28). These are three highly relevant implementation concept points to cover early in an implementation process. Particularly the implementation concept point of supervisor support has been shown to be important for implementation of workplace initiatives (20, 29). However, to become successful with the implementation of a new action it is important to work with all 11 implementation concept points (19), and none of the workplaces had all implementation concept points covered. Still, the majority of the adopters did not return to the checklist. One reason may be that they did cover the remaining implementation concept points after the first usage of the checklist, but did not have the incitements to fill in the checklist again. Another potential explanation for the lack of returning users may be found in those implementation concept points that the workplaces generally did not cover by the first time of usage. For example, lack of resources allocated was one of the most frequent implementation concept points not dealt with by the first usage. Lack of resources may disrupt the entire progress of the implementation and thus explain the low number of returning users (30). Another implementation concept point with low coverage was the involvement of all relevant employees (31). Involvement of many employees in participatory processes is shown to be highly demanding on organizations and may have disrupted further implementation (26). Overall, it is likely, that some workplaces found usage of the checklist and actually obtained the implementation, they expected. However, it is also likely, that some implementation processes were disrupted due to the checklist making the workplaces aware of the high demands for implementation of new habits. Ultimately, the checklist may help workplaces quit unrealistic actions and focus on smaller, more implementable actions.

### What Is the Effectiveness?

Considering the relatively low implementation of the checklist, it would be unrealistic to expect a large effect. Furthermore, those who were reached by the campaign scored higher at baseline on the prioritization of the work environment compared to those not reached. At follow-up, both groups reported a non-significant decrease in prioritization of the work environment. Data from a national Danish work environment survey conducted in 2012, 2014, 2016, and in 2018 also reporting the prioritizing of the work environment from the eldercare sector indicate the same overall decreasing of the prioritizing in the period (32). Overall, it is challenging to disentangle the effect of behavioral interventions in observational designs, as the effectiveness and *real life* impact of the intervention (in this project the effect of using the checklist in implementing new routines in the work environment) is highly sensitive to so many factors that cannot be directed in an evaluation, particularly not when implementation is low.

### Strengths and Limitations

We used all five components in the RE-AIM framework to evaluate the impact of the checklist, which is a strength of the study. Evaluation of a dissemination project like this is complex, and therefore the RE-AIM framework was the best suited framework for guiding the evaluation. Because of the complexity it is difficult to separate the effect of all the activities and therefore the overall impact as stated earlier is also difficult to highlight as one final quantity. A strength of the study was the systematic dissemination strategy using a large variation of dissemination channels, and to ensure a sustainable change we involved a broad range of relevant stakeholders in the eldercare sector. The involvement of stakeholder resulted in a large support for the campaign activities, large dissemination, but insufficient implementation. Finally, another strength of this study was the use of multiple data sources not only using self-reported data.

Limitations are that we were unable to evaluate “offline” usage of the checklist, which was also part of the campaign. Furthermore, campaign intensity was highest in the first 3 months and the evaluation time was only 12 months. Prolonging the intense campaign activities and the evaluation period and supporting the maintenance/returning users would have increased the relevance of effect evaluation and a more full impact evaluation. Another limitation is, that the many different dissemination strategies couldn't be measured. This could have actually made the results appear less robust than they might have been. A limitation for adoption, is that we could not connect all 534 accounts to a specific workplace to see whether they all were eligible workplaces. So this means that our adoption might be underestimated. In addition to this, users who are reported as reached could actually also be those who are reported in adoption. Adoption is meant to represent organizational uptake of the tool. However, with one registered user in one organization, the data cannot reveal whether the user represents the entire organization (i.e., cooperating with other members of the health and safety organization) or if the user operates singlehandedly. This means that our measure of adoption may be biased—both possibly underestimated because all users couldn't be matched to a certain workplace, but also likely overestimated as a measure of adoption, because some users may not have implemented the tool organizationally. Our study of adoption should therefore be considered in the light of this limitation.

### Implications

This study contributes to the field of research to practice or knowledge translation. First, it constitutes an example of how to disseminate and translate research knowledge to a relatively large fraction of the nursing homes in Denmark. Furthermore, it gives input on what the implementation concept points that may hinder implementation in the eldercare workplaces are. Finally, it maps out useful communication channels in the sector and topics for action that are top of mind in the adopting workplaces. Disemmination strategies are difficult to track and measure. Future research should consider innovative ways to track user-journeys between dissemination efforts and usage, i.e., through interviews of the users or by various kinds of digital footprints.

## Conclusion

In conclusion, this study shows that a 1-year stakeholder-supported national campaign can disseminate knowledge to a large number of workplaces in the Danish eldercare sector. Useful dissemination channels are those, which the target population already trusts, and access for information. Implementation of the checklist was not satisfactory, and good implementation may require a certain level of organizational readiness for change. Usage of the checklist may reveal that implementation is more demanding than expected by Danish eldercare workplaces.

## Data Availability Statement

The datasets generated for this study are available on request to the corresponding author.

## Informed consent

By law, no informed consent is needed when using survey data.

## Ethics Statement

Ethical review and approval was not required for the study on human participants in accordance with the local legislation and institutional requirements. Written informed consent for participation was not required for this study in accordance with the national legislation and the institutional requirements.

## Author Contributions

PM handled the data and did the analyses, drafted the first version of the manuscript, and wrote the final version of the manuscript. MJ acquired funding for the project, designed the study, and participated in discussions around the study, and critically revised the manuscript. HH designed the study and participated in discussions around the study, and critically revised the manuscript. CR acquired funding for the project, designed the study, and critically revised the manuscript. All authors have read and approved the manuscript. All authors contributed to the article and approved the submitted version.

## Conflict of Interest

The authors declare that the research was conducted in the absence of any commercial or financial relationships that could be construed as a potential conflict of interest.
